# The Influence of Nordic Walking on Spinal Posture, Physical Function, and Back Pain in Community-Dwelling Older Adults: A Pilot Study

**DOI:** 10.3390/healthcare9101303

**Published:** 2021-09-30

**Authors:** Yi-Hung Huang, I-Yao Fang, Yi-Liang Kuo

**Affiliations:** 1 Department of Orthopedics, Ditmanson Medical Foundation Chia-Yi Christian Hospital, Chiayi City 600, Taiwan; cychaudiofan@gmail.com; 2 Department of Orthopedics, National Cheng Kung University Hospital, Tainan City 704, Taiwan; 3 Physical Education Center, Southern Taiwan University of Science and Technology, Tainan City 710, Taiwan; fyy125@gmail.com; 4 Department of Physical Therapy, College of Medicine, National Cheng Kung University, Tainan City 701, Taiwan

**Keywords:** aging, walking, kyphosis, spine, physical fitness, back pain, muscle performance

## Abstract

Nordic walking is an increasingly popular form of exercise among the elderly. Using poles is thought to facilitate a more upright posture; however, previous studies primarily investigated the effects of Nordic walking on respiratory function and physical fitness. The aims of this study were to investigate the influence of Nordic walking on spinal posture, physical functions, and back pain in community-dwelling older adults. Thirty-one community-dwelling older adults aged ≥ 60 years participated in a twice weekly Nordic walking training program for 12 weeks. The outcome measures, including spinal posture, physical functions, back pain, and the strength and endurance of back extensor muscles were assessed before and after a 12-week program. After training, spinal posture, back pain, and the strength and endurance of back extensor muscles did not show any statistically significant changes. Among the seven clinical tests of physical function, only the 30 s arm curl test, the 30 s chair stand test, and the single leg stance test showed significant improvements. Nordic walking has limited influence on age-related hyperkyphosis and back pain, but may be effective for physical function. The results of this study can provide useful information for people involved in the prevention and treatment of physical dysfunction in community-dwelling older adults.

## 1. Introduction

The prevalence of thoracic hyperkyphosis is estimated to be between 20% and 40% among older adults aged 60 and over [1]. Normally, the thoracic spine presents with a small amount of kyphotic curvature because of the shape of the vertebral bodies and intervertebral discs [2]. The mean thoracic kyphosis in people under 40 years old is between 20°–29° [3]. After late middle age, the kyphotic angle tends to rapidly increase, especially in women [4].

Increased thoracic kyphosis is associated with several significant health consequences, including back pain, impaired physical function, reduced lung function, impaired balance, increased incidence of falls and future fracture risk, decreased quality of life, and increased mortality [2,3]. Ordu et al. found that patients with pathologic kyphosis > 40° had significantly lower forced vital capacity and respiratory rate than those with normal physiologic kyphosis ≤ 40° [5]. Kado et al. reported significantly a higher percentage of hyperkyphotic posture among older adults who fell than those who did not fall (36.3% versus 30.2%, *p* = 0.015) [6]. In a following study, Kado et al. reported that older adults with even slight hyperkyphosis (the occiput-to-table distance ≥ 1.7 cm when lying supine on an examination table) had a 1.44 times greater rate of mortality than those without hyperkyphotic posture [7].

Many risk factors are thought to contribute to hyperkyphosis in older adults. The most recognized risk factors are vertebral fractures and osteoporosis [2]; however, one early study suggests that vertebral fractures only explained about 42–48% of kyphosis variance [8]. The degenerative change of the anterior longitudinal ligament is another proposed risk factor [1]. The decreased elasticity of connective tissues can affect the ability to attain an upright posture and contribute to an increase in the Cobb angle of kyphosis. Back extensor strength has also been shown to be negatively associated with the kyphosis angle in older women [9,10]. Mika et al. examined thoracic kyphosis, back extensor strength, and bone mineral density in 189 women aged 50–80 years [9]. The result of multivariate analyses showed that only back extensor strength might influence thoracic kyphosis.

Many exercise-based interventions have focused on modifiable risk factors such as muscle strength and flexibility in order to rehabilitate age-related hyperkyphosis in older adults [11,12,13,14,15]. Nordic walking is a particular form of walking, and involves the use of specially designed walking poles in opposition to lower limb locomotion. From a biomechanical perspective, using the poles is thought to facilitate a more upright posture; however, there are only limited scientific reports examining the effect of Nordic walking on spinal posture in older adults [16,17,18]. The results are inconsistent, and none of the studies used radiographic images to assess thoracic kyphosis.

As the population rapidly ages, the prevalence of age-related hyperkyphotic posture and spinal pain in older adults are expected to increase. If left untreated, hyperkyphosis may cause physical impairments and dysfunction as mentioned above, and impact older adults’ general health. Nordic walking engages the whole body and also provides extra stability during walking. Once someone overcomes the poling technique, Nordic walking may be a safe and alternative exercise option to minimize the progression and negative consequences of age-related hyperkyphosis. Nordic walking can also provide health benefits for physical activity, such as improving maximum heart rate, peak oxygen consumption, and body composition [19,20]. Therefore, the primary aim of this pilot study was to investigate the influence of Nordic walking on spinal posture, physical function, and back pain in community-dwelling older adults. The strength and endurance of back extensor muscles were also measured before and after Nordic walking training.

## 2. Materials and Methods

### 2.1. Study Design

This was a single-group pretest-posttest study. The participants performed a 12-week Nordic walking training program, and were instructed not to change their usual physical activity. Data collection was conducted at the university’s laboratory and hospital. This study was approved by the Institutional Review Board of National Cheng Kung University Hospital (A-BR-108-015) and conducted in compliance with the Declaration of Helsinki.

### 2.2. Participants

A convenient sample of community-dwelling older adults was recruited from the local communities around the university campus by flyers and from the relatives and friends of the participants. The inclusion criteria were: (1) aged 65 or above; (2) able to communicate and understand testing and exercise instructions; (3) able to walk unaided (i.e., without a cane or walker); (4) have no range of motion limitation in the upper extremities affecting the use of walking poles; (5) have no serious and limiting health conditions restricting exercise participation. The exclusion criteria were: (1) cognitive impairment (Mini-Mental State Examination < 23) [21]; (2) reported painful vertebral fractures in the previous 3 months; (3) reported total hip or knee replacement or lower extremity fracture within the previous 6 months; (4) reported unexplained weight loss > 4.5 kg or ≥ 3 falls in the previous year; (5) reported advanced disability or end-stage disease, major psychiatric illness, vestibular or progressive neurologic disorder; (6) inability to pass safety tests for exercise participation at the screening testing (gait speed < 0.6 m/s, inability to stand with feet together for 30 s, inability to actively flex shoulders to 90°, and inability to move from standing to supine position on a mat and return to standing independently or with the use of a nearby chair) [22].

**Figure 1:**
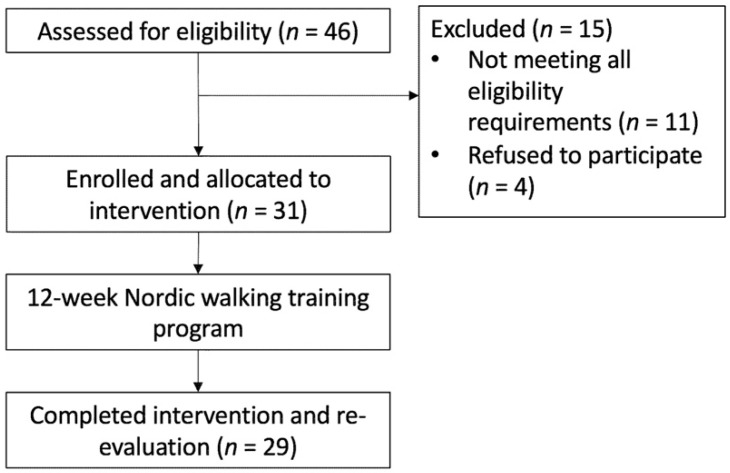
Flow diagram of participant recruitment and retention.

Once the preliminary study criteria were prescreened by telephone, a face-to-face screening was scheduled for safety tests. When the participants came into the laboratory, they were provided with an information sheet and informed about the purpose and procedures of the study in details. If the participants met all the inclusion and exclusion criteria, the participants then signed an informed consent to participate in the study (Figure 1).

### 2.3. Intervention

The Nordic walking training program was conducted twice a week for 12 weeks and supervised by a qualified Nordic walking instructor and a trained assistant. The participants were instructed not to take up any additional forms of physical activity, and not to change their current eating and exercise habits during the training period.

**Figure 2:**
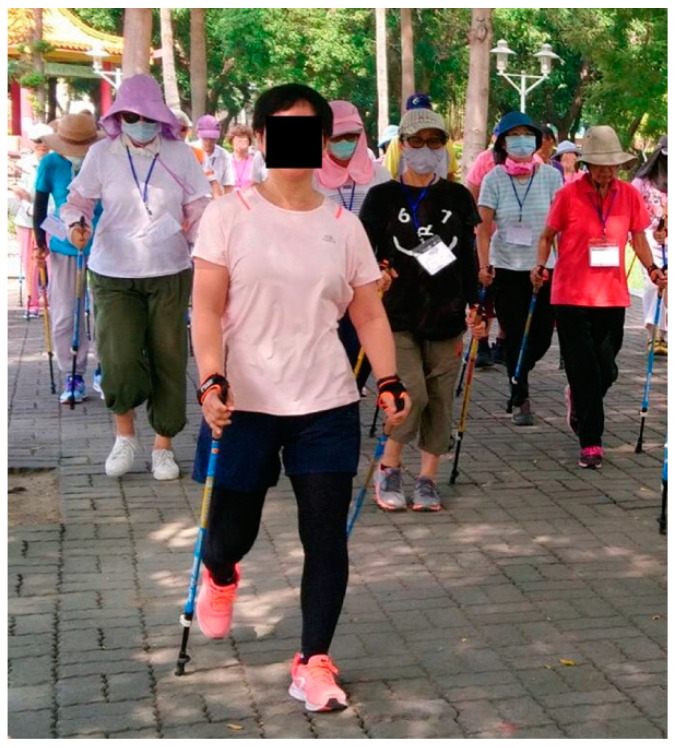
Nordic walking.

The Nordic walking training program was created by qualified Nordic walking instructors and conducted in a city park (Figure 2). Each class comprised approximately 10 min of warm-up exercises, 20 min of strengthening exercises, 30 min of Nordic walking, 20 min of aerobic and balance exercises, and 20 min of cool-down exercises [19] (Table S1). The warm-up exercises included gentle stretching and breathing that gradually adjusts the participants to a higher intensity of exercise and reduces the risk of injuries. Strengthening exercises were performed for the major muscle groups in the upper and lower extremities with the poles and elastic resistance bands. For the main exercise, the participants were instructed to the proper poling technique first and then walked around the park with the poles. Considering that the participants were elderly, the exercise intensity was set by each participant’s subjective rate of perceived exertion. The participants were encouraged to gradually increase their walking distance and pace during the training period. After the main exercise, step exercises, intermittent running in place, or jumping were performed to improve aerobic capacity and coordination, and tandem or single leg stance were performed to improve dynamic balance. Finally, cool-down exercises were performed for stabilizing and restoring normal cardiorespiratory function.

### 2.4. Outcome Measures

The participants performed the same set of tests at the beginning and end of the training. The primary outcome measures included spinal posture (thoracic kyphosis angle and sagittal alignment), physical function, and back pain. The secondary outcome measures were the strength and endurance of back extensors.

#### 2.4.1. Thoracic Kyphosis Angle

The thoracic kyphosis angle was assessed using the occiput-wall distance (OWD) [23]. The OWD is a simple measure used to screen thoracic kyphosis in clinical settings, and had good concurrent validity with the Cobb angles (r = 0.68, *p* < 0.001) [23]. A rigid ruler was used to measure the distance between the occiput and the wall while the participant was standing with both heels and the sacrum against the wall, maintaining a horizontal gaze. The measurement was repeated twice, first in the usual standing posture, and then in the best standing posture.

#### 2.4.2. Sagittal Alignment

**Figure 3:**
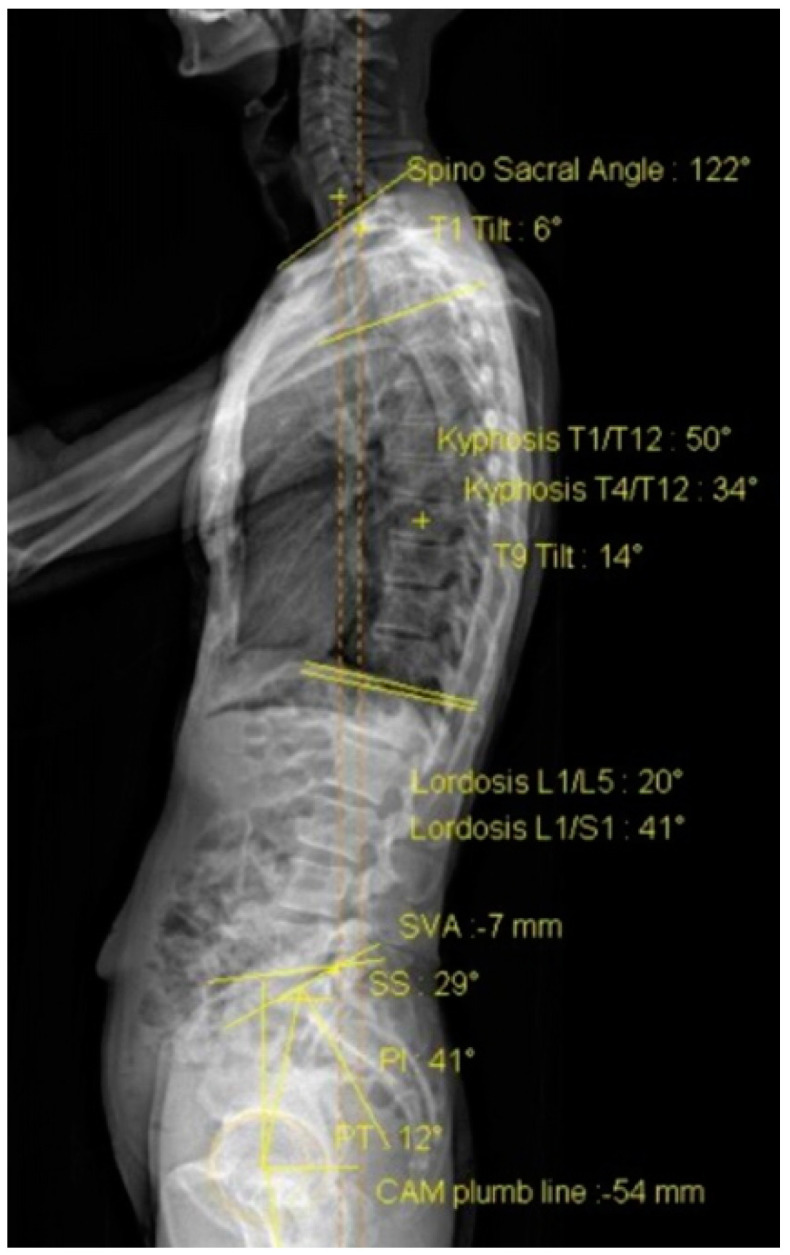
EOS imaging. PI: pelvic indigence; SS: sacral slope; PT: pelvic tilt; SVA: sagittal vertical axis; CAM: center of the acoustic meatus.

The sagittal alignment was assessed using the EOS^TM^ imaging system [24]. The EOS^TM^ imaging system utilizes 2-dimesional X-rays acquired in an upright position technology to reconstruct a 3-dimesional full spine model. A previous study reported that the EOS^TM^ imaging system has excellent intra-rater (ICC 0.90 to 0.98) and inter-rater (ICC 0.79 to 0.84) reliability for assessment of the sagittal alignment of the spine [25]. The standardized imaging procedure was used, with the participant standing upright and arms folded at 45° to reduce superimposition on the spine. The following measurements were used for data analysis (Figure 3) [25]: the angle between the superior endplate surface of the T4 and the inferior endplate surface of the T12 (kyphosis T1/T12); the angle between the superior endplate surface of the L1 and the inferior endplate surface of the L5 (lordosis L1/L5); the angle formed by a line drawn between the center of the femoral head and the sacral endplate (pelvic incidence, PI); the angle between a horizontal line and the slope of the superior sacral endplate surface (sacral slope, SS); the angle between the vertical plumb line from the femur head center and the center point of the superior sacrum endplate surface (pelvic tilt, PT); the distance from the plumb line from the center of the C7 to the posterior edge of the upper sacral endplate surface (sagittal vertical axis, SVA).

#### 2.4.3. Physical Function

Physical function was assessed using the functional fitness test [26]. The test includes measures of upper and lower body strength (the 30 s arm curl test and the 30 s chair stand test), upper and lower body flexibility (the back scratch test and the chair sit and reach test), aerobic endurance (the 2 min step test), and balance (the 2.44 m up-and-go test and the single leg stance test). For the 30 s arm curl and chair stand tests, the number of lifts or stands during 30 s was recorded. For the back scratch test and the chair sit and reach tests, the best distance (cm) of two trials was measured by a ruler. For the 2 m step test, the number of steps in a single trial was recorded. For the 2.44 m up-and-go test and the single leg stance test, the best time (sec) of two trials was measured by a stopwatch.

#### 2.4.4. Back Pain

Back pain in the previous week was assessed using a 11-point horizontal numeric rating scale (NRS) with terminal descriptors of 0 (no pain) and 10 (worst pain possible). The NRS has established clinimetric properties in low back pain, and a change of ≥2 points represents a clinically meaningful improvement [27].

#### 2.4.5. Strength and Endurance of Back Extensor Muscles

Back extensor strength was measured in the sitting position using a microFET2 dynamometer (Hoggan Health Industries Inc., Salt Lake City, UT, USA) [9]. The participant was instructed to sit with the hips and knees flexed at 90° and arms crossed on the chest. The upper edge of the dynamometer was aligned with the superior borders of the scapulae across the midline. The participant was instructed to extend the trunk backward with maximal effort. The maximum record in three trials was recorded.

Back extensor endurance was assessed using the Timed Loaded Standing test [13]. This test is safe for older adults with vertebral osteoporosis, and simulates functional performance of the trunk during daily activities. The test demonstrates good test-retest reliability (ICC = 0.84) and acceptable concurrent validity with functional performance (Spearman’s rho = 0.52) [28]. The participant was instructed to hold a 2 lb. dumbbell in each hand with the arm maintained at 90° of shoulder flexion and elbow extended. The holding time was measured, and the average of two trials was calculated for data analysis.

### 2.5. Statistical Analysis

The statistical analysis was conducted using the statistical software SPSS (version 21.0, IBM, New York, NY, USA). Continuous variables were checked by the Shapiro-Wilk tests for normality. The descriptive statistics were summarized for all demographics and dependent variables. The normally distributed data was expressed as a mean and standard deviations (SD), and non-normally distributed data was expressed as a median and interquartile range (IQR). A Wilcoxon signed rank test was used to analyze the difference in back pain before and after the training. A paired t-test was used to analyze the difference in the other dependent variables. The effect size (d) was calculated and interpreted according to the benchmarks provided by Cohen: small (d = 0.2), medium (d = 0.5), and large (d = 0.8) effects [29]. The level of statistical significance was set at *p* < 0.05.

## 3. Results

Forty-six community-dwelling older adults were initially screened for eligibility, and 31 people (9 men, 24 women; median age 71 years, IQR 61–81 years; mean height 157.3 cm, SD 7.7 cm; mean weight 57.4 kg, SD 11.7 kg; median body mass index 23.4 kg/m^2^, IQR 19.0–27.8 kg/m^2^) met the inclusion and exclusion criteria and agreed to participate. One participant underwent surgery for a chronic condition and could not complete the training. Her husband decided to drop out at the same time. This resulted in a total of 29 participants for data analysis. Table 1 summarizes the characteristics of participants at baseline. The participants completed a minimum of 19 out of 24 classes, and the average completed classes are 23.0 ± 1.5.

Table 2 summarizes the outcome measures before and after the training. There was no significant difference in the thoracic kyphosis angle, sagittal alignment, and strength and endurance of back extensor muscles (*p* > 0.05). A statistically significant improvement was noted in some measures of physical function, specifically the 30 s arm curl test (95% confidence interval [CI] [0.10, 2.32], *p* = 0.034), the 30 s chair stand test (95% CI [0.12, 1.74], *p* = 0.026), and the single leg stance test (95% CI [2.80, 9.41], *p* = 0.001).

**Figure 4:**
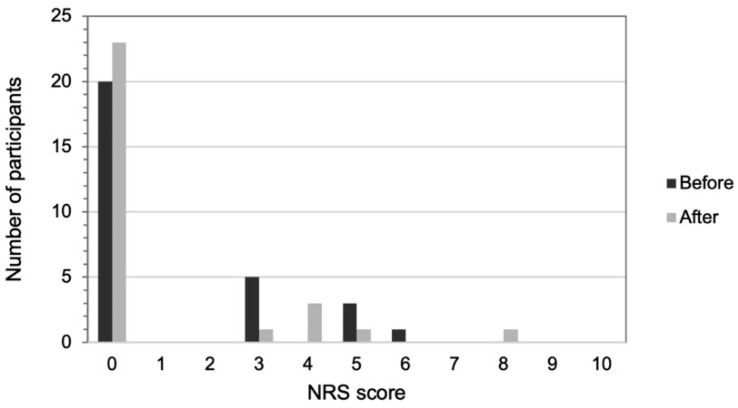
The distribution of numeric rating scale (NRS) scores for lower back pain before (black bars) and after (gray bars) the Nordic walking training program.

None of the participants reported any upper back pain before or after training. Figure 4 illustrates the distribution of the NRS score for lower back pain. Among nine (31%) participants with lower back pain before training, six participants reported improvement in the NRS score, two participants reported worsening, and one reported unchanged after training. The Wilcoxon signed rank test showed no significant difference in the NRS scores for lower back pain before and after the training program (Z = −0.81, *p* = 0.421).

## 4. Discussion

Nordic walking is an increasingly popular form of exercise for older adults. The aim of this pilot study was to determine the influence of Nordic walking on the improvement of spinal posture, physical function, back pain, and the strength and endurance of back extensor muscles in community-dwelling older adults. The results showed that a 12-week Nordic walking training program did not significantly improve spinal posture, back pain, or the strength and endurance of back extensor muscles. Among seven clinical tests of physical function, only the tests for upper and lower body strength and balance showed improvement.

### 4.1. Spinal Posture

Nordic walking requires the forward inclination of the trunk, resulting in a smaller trunk flexion angle [16] and increased activation of the trunk muscles [30]. However, there are limited scientific reports examining the effect of Nordic walking on spinal posture in older adults [16,17,18]. The results of insignificant changes of spinal posture in this study are consistent with two previous studies, which found that 6–8 weeks of Nordic walking training did not significantly alter spinal posture during walking in healthy, older adults [16] and elderly women with Parkinson’s disease [18]. In contrast, a previous study showed that, compared to general gymnastics, eight weeks of Nordic walking training significantly reduced thoracic kyphosis of middle-aged women after breast cancer treatment [17].

Several reasons may account for the inconsistent findings on spinal posture between multiple studies. First, according to the diagnostic cut-off point of 6.5 cm with the OWD test [23], only 10 participants in this study were considered to have thoracic hyperkyphosis. Therefore, the insignificant changes in spinal posture might be largely due to the ceiling effect. Second, the duration and format of exercise-based intervention can influence the effectiveness of Nordic walking training. A previous study suggested that at least eight weeks is necessary to acquire the poling technique and gain possible benefits [16]. Although the duration of 12-week Nordic walking training in this study should have been sufficient, group training might have had some disadvantages. It may have been challenging for the instructor to ensure that every participant performed the poling technique perfectly and also achieved individual, optimal exercise intensity. Moreover, a previous study found that the significant correlation between thoracic kyphosis and back extensor strength only existed in women with weak back extensor strength < 35 N [9]. A loss of back extensor strength can compromise the capability of generating spinal extension movement, which leads to an increase in the kyphotic angle. The insignificant changes of spinal posture in this study may be related to the baseline characteristics of participants and insignificant improvement in the strength and endurance of back extensor muscles.

Thoracic hyperkyphosis is a commonly recognized postural problem in older adults; however, evidence on the age-related deterioration of spinal posture for both genders is controversial. Uehara et al. reported a gender difference in spinal alignment deviation in a cohort of 413 older adults (50–89 years old) who were randomly sampled from the resident registry of a rural area in Japan [31]. Although the global spinal alignment deviated anteriorly with age for both genders, cervical protrusion was noticeable from a younger age in men and decreased lumbar lordosis and sacral slope occurred earlier in women. The results of a 4-year longitudinal cohort study conducted by Oe et al. in Japan also support gender differences in changes of the spinal posture; however, no significant deterioration was found in men’s cervical spine alignment and women’s lumbar lordosis [32]. This study recruited both men and women. Analyzing the data as a whole may have contributed to the insignificant findings in spinal posture.

### 4.2. Physical Function

The results of this study support the literature that Nordic walking as a mean of physical activity provides health benefits for community-dwelling older adults [19,20,33]. In this study, 12 weeks of Nordic walking training led to an increase in upper and lower body strength by 7.1% and 5.4%, respectively. Balance as measured by the single leg stance test with eyes open also improved by 30.9%. However, these effect sizes were smaller than those values reported by Bullo et al. using the same training duration and measurement method (upper body muscle strength, 11.6–19.7%; lower body strength, 9.5–25.9%; balance, 133.9%) [19]. Furthermore, in contrast to their results [19], 12 weeks of Nordic walking training in this study did not show any significant improvement in flexibility and aerobic endurance. The difference between studies may be partly due to the different characteristics of participants. Previous studies of Nordic walking targeted frail [34] or sedentary [35,36] older adults, while some participants in this study were already physically active and engaged in structured exercises at least two to three times a week.

### 4.3. Back Pain, Back Extensor Strength, and Back Extensor Endurance

The associations between hyperkyphosis and either back pain or strength and endurance of back extensor muscles were reported in previous studies [2,3]. The insignificant findings in back pain, back extensor strength, and back extensor endurance in this study likely resulted from no significant changes in spinal posture. Progressive loading and specificity are key determinants of effective resistance training [37]. Previous exercise studies targeting age-related hyperkyphosis included back extensors exercises such as prone trunk extension and arm lift standing against wall, and also used equipment such as dumbbells, resistance bands, or weighted backpack [15]. Compared with previous studies, isolated trunk exercise was not performed in this study. The effort of maintaining an upright posture during Nordic walking might not be challenging enough for increasing strength and endurance of back extensor muscles; therefore, positive results reported in previous studies [12,38] were not found in this study.

### 4.4. Limitation and Strength

There were several limitations in this study. First, a single-group pretest-posttest study does not allow comparison of the effect of Nordic walking training to walking without poles. In addition, some participants had medical diseases and conditions such as osteoporosis, knee arthritis, or Parkinson’s diseases, which may have influenced the results. This study was conducted under the impact of COVID-19. The concern of getting sick seriously influenced the willingness of community-dwelling older adults to participate in a group exercise program. The influence of the pandemic also resulted in the decision to cancel the follow-up assessment. Second, most participants in this study were physically active and did not have severe thoracic hyperkyphosis, which may have limited the effectiveness of the program, and also influenced a generalization of the results. The strength of this study is that, in addition to clinical examination (the OWD test), spinal posture was assessed by the new EOS^TM^ imaging system. The use of EOS images allows radiographic measurements with greater accuracy and reliability, and also provides information about sagittal balance and pelvic parameters. Further study using a randomized controlled trial and recruiting a larger group of community-dwelling older adults with more severe thoracic hyperkyphosis will be required to determine the definitive effects of Nordic walking on spinal posture and back pain.

## 5. Conclusions

Based on our preliminary findings, Nordic walking has limited influence on the improvement of spinal posture and back pain in community-dwelling older adults. However, a 12-week Nordic walking training program has a potential to improve upper and lower body strength and balance. The results of this study can provide useful information for people involved in the prevention and treatment of physical dysfunction in community-dwelling older adults. Future studies are needed to clarify the effects of Nordic walking in older adults with age-related hyperkyphosis.

## Figures and Tables

**Table 1:** Baseline characteristics of study participants (*n* = 29).

Variable	All Participants
Gender	8 men, 23 women
Age (year)	70 (8)
Height (cm)	157.2 (8.0)
Weight (kg)	57.2 ± 12.0
Body mass index (kg/m^2^)	22.6 (4.6)
Mini-Mental State Examination (score)	29 (3)
High blood pressure	8/34.5%
Diabetes	5/17.2%
Osteoporosis	7/24.1%
Knee arthritis	5 (17.2%)
Heart diseases	5 (17.2%)
Parkinson disease	1 (3.4%)
Stroke	1 (3.4%)
*Education*	
Primary school and illiterate	4/13.7%
High school	10/34.5%
College and above	15/51.7%
*Marital Status*	
Married	23/79.3%
Single, widowed, and divorced	6/20.7%
*Physical Activity Level ^1^*	
High	11/37.9%
Moderate	17/58.6%
Low	1/3.4%

Note. Data are presented as mean ± standard deviations, median (interquartile range), or number/percentage. ^1^ Categorized according to the results of the International Physical Activity Questionnaire.

**Table 2:** Changes in outcome measures before and after the pole walking exercise program.

Outcome Measures	Pre-Test	Post-Test	Δ	*p*	d
*Thoracic kyphosis angle*					
OWD (usual, cm)	6.79 ± 3.64	6.44 ± 2.53	−0.35	0.419	0.15
OWD (best, cm)	4.86 ± 4.81	4.81 ± 2.15	−0.04	0.917	0.02
*Sagittal alignment*					
T1/T12 kyphosis (°)	44.31 ± 9.80	44.42 ± 10.03	−0.12	0.891	0.03
L1/L5 lordosis (°)	26.04 ± 14.99	25.54 ± 14.79	0.50	0.383	0.17
Pelvic incidence (°)	48.50 ± 12.05	48.04 ± 12.07	0.46	0.474	0.14
Sacral slope (°)	31.27 ± 9.41	31.31 ± 9.37	−0.04	0.952	0.12
Pelvic tilt (°)	17.12 ± 8.09	16.81 ± 8.34	0.31	0.415	0.16
Sagittal vertical axis (mm)	36.04 ± 27.06	40.38 ± 26.10	−4.35	0.204	0.26
*Physical function*					
30-s biceps curl (time)	17.07 ± 3.67	18.28 ± 3.74	1.21	0.034 *	0.41
30-s chair stand (time)	17.07 ± 4.54	18.00 ± 4.33	0.93	0.026 *	0.44
Back scratch (cm)	1.37 ± 9.72	1.97 ± 8.23	0.60	0.400	0.16
Chair sit and reach (cm)	9.84 ± 17.34	7.98 ± 17.28	−1.86	0.051	0.38
2-min step (time)	95.62 ± 21.27	98.93 ± 19.82	3.31	0.308	0.19
2.44-m up-and-go (s)	5.83 ± 1.38	5.86 ± 1.16	0.03	0.798	0.05
Single leg stance (s)	19.74 ± 11.48	25.85 ± 7.76	6.10	0.001 *	0.70
Back extensor strength (N)	39.27 ± 7.83	39.52 ± 7.44	0.25	0.855	0.03
Back extensor endurance (s)	148.55 ± 91.37	170.33 ± 115.10	21.79	0.053	0.38

Note. Data are presented as mean ± standard deviations. OWD = occiput-to-wall distance. **Δ** = mean difference. d = effect size. * *p* < 0.005.

## Data Availability

The data supporting the conclusion of this article will be made available upon request from the corresponding author.

## Supplementary Materials

The following are available online at https://www.mdpi.com/article/10.3390/healthcare9101303/s1, Table S1: 12 weeks of Nordic walking training program.
