# Supplementary material for: The Influence of Nordic Walking on Spinal Posture, Physical Function, and Back Pain in Community-Dwelling Older Adults: A Pilot Study
Source: Healthcare (Basel). 2021 Sep 30;9(10):1303. doi: 10.3390/healthcare9101303 (PMC8544539; doi:10.3390/healthcare9101303)
Supplement: Supplementary file 1 [file healthcare-09-01303-s001.zip › healthcare-1360024-supplementary.pdf]

Table S1. 12 weeks of Nordic walking training program

Session	Intensity by RPE		Exercises
	Week 1-6	Week 7-12	
Warm-up (10 min)	3-4	3-4	Light step aerobics (3-5 min)<br>Full body stretching (5 min)
Pole strengthening (20 min)	3-4	5-6	Chest, shoulder, back, arm, abdominal muscles and leg exercises<br>10 repetitions, 2 sets, with elastic band
Nordic walking (30 min)	3-4	5-6	Normal speed (10 min) → fast speed (10 min) → normal speed (10 min)
Pole aerobic and balance (20 min)	3-4	5-6	Pole aerobic exercises (10 min)<br>Pole balance exercises (10 min)
Cool-down (20 min)	3-4	3-4	Full body stretching (20 min)

Note. RPE = Rating of Perceived Exertion (scale 0-10)
